# Supplementary material for: Effects of Trichoderma harzianum combined with Phanerochaete chrysosporium on lignin degradation and humification during chicken manure and rice husk composting
Source: Front Microbiol. 2025 Feb 28;16:1515931. doi: 10.3389/fmicb.2025.1515931 (PMC11906335; doi:10.3389/fmicb.2025.1515931)
Supplement: Supplementary file 1 [file Data_Sheet_1.zip › 6.Function/CKS_BFHS/FunGuild/l2/metastats/Animal_Endosymbiont.CKS_BFHS.metastats.pdf]

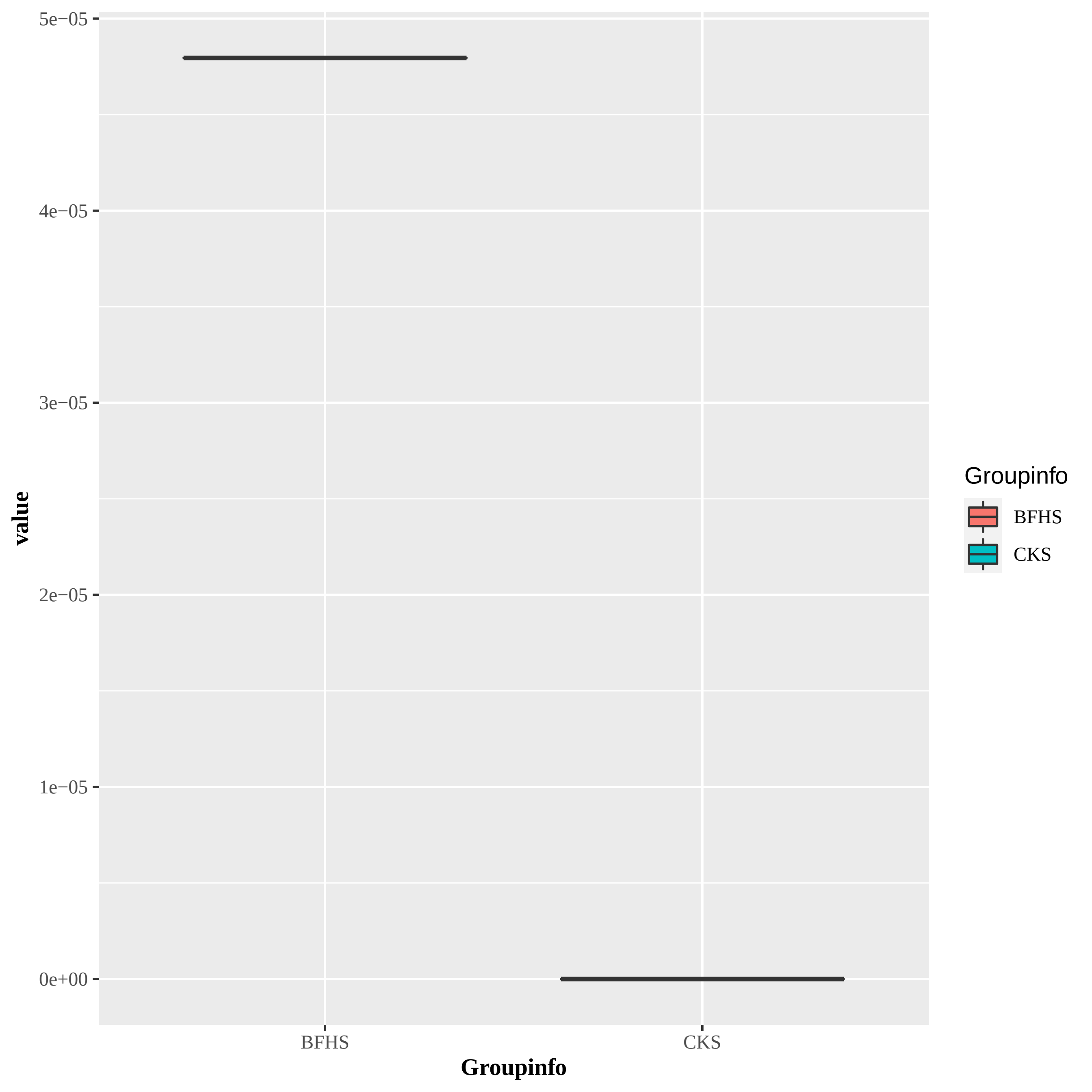
5e−05
4e−05
3e−05
2e−05
1e−05
0e+00
value
BFHS
CKS
Groupinfo
Groupinfo
BFHS
CKS
